# Hydroxynaphthoquinone Metal Complexes as Antitumor Agents X: Synthesis, Structure, Spectroscopy and *In Vitro* Antitumor Activity of 3-Methyl-Phenylazo Lawsone Derivatives and Their Metal Complexes Against Human Breast Cancer Cell Line MCF-7

**DOI:** 10.1155/MBD.2000.121

**Published:** 2000

**Authors:** Nikhil Gokhale, Subhash Padhye, Chris Newton, Robin Pritchard

**Affiliations:** 1 Department of Chemistry University of Pune Pune 411 007 India; 2 Department of Medicine University of Hull Hull HU6 7RX UK; 3 Department of Chemistry UMIST Manchester M60 10QD UK

## Abstract

The C-3 substituted phenylazo derivatives of lawsone (2-hydroxy-l,4 p-naphthoquinone, III) were synthesized and characterized. The X-ray crystal structure was determined for the ligand 3-(3′-methyl phenylazo) lawsone. The copper complexes of these derivatives were found to possess 1:2 metal stoichiometry and square planar geometries with intermolecular stackings, resulting in antiferromagnetic exchange interactions. The *in vitro* activity of all the synthesized compounds was examined against human breast cancer cell-line, MCF-7, which revealed enhanced activities for the metal complexes, the highest activity being observed for the copper compound of 3-(3′-methyl phenylazo) lawsone.


### HYDROXYNAPHTHOQUINONE METAL COMPLEXES AS ANTITUMOR AGENTS X: SYNTHESIS, STRUCTURE, SPECTROSCOPY AND *IN VITRO* ANTITUMOR ACTIVITY OF 3-METHYL -PHENYLAZO LAWSONE DERIVATIVES AND THEIR METAL COMPLEXES AGAINST HUMAN BREAST CANCER CELL LINE MCF-7

Nikhil Gokhale[1], Subhash Padhye*[1], Chris Newton[2], and Robin Pritchard[3]

[1]Department of Chemistry, University of Pune, Pune-411 007, India.
[2]Department of Medicine, University of Hull, Hull HU6 7RX, UK.
[3] Department of Chemistry, UMIST, Manchester, M60 10QD, UK.

**Abstract**: The C-3 substituted phenylazo derivatives of lawsone (2-hydroxy-1,4 p-naphthoquinone, III) were synthesized and characterized. The X-ray crystal structure was determined for the ligand 3-(3′-methyl phenylazo)lawsone. The copper complexes of these derivatives were found to possess 1:2 metal stoichiometry and square planar geometries with intermolecular stackings, resulting in antiferromagnetic exchange interactions. The *in vitro* activity of all the synthesized compounds was examined against human breast cancer cell-line, MCF-7, which revealed enhanced activities for the metal complexes, the highest activity being observed for the copper compound of 3-(3′-methyl phenylazo) lawsone.

### Introduction:

Quinonoidal ligands when used as therapeutic agents can exercise their cytotoxic action by various mechanisms which include redox cycling, arylation, intercalation, induction of DNA strand breaks, generation of free radicals and alkylation via quinone methide formation[1,2]. The ability of 1,4 naphthoquinone to produce ‘oxidative stress’ by redox cycling and to act preferentially as alkylating agents is well established[3,4]. The representative examples of this class of compounds include well-known anticancer drugs of the anthracycline and naphthazarin series[5,6]. Analogous ligands like plumbagin (**I**) and shikonin (**II**) are also attributed with anticancer activity[7,8]. Recently it has been shown by Couladouros et al.[9] that the 1,4 naphthoquinones bearing at least one phenolic hydroxyl group are potent inhibitors of topoisomerase enzymes. The ability of these ligands to complex divalent metal ions such as $Zn^{+2}$, $Cu^{+2}$,$Mg^{+2}$ parallels their topoisomerase inhibition property and is thought to be the underlying mechanism of their anticancer activity.

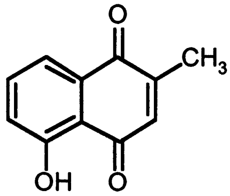

(I)

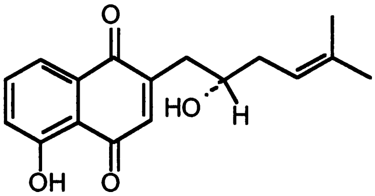

(II)

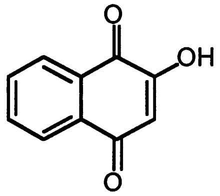

(III)

Lawsone (2-Hydoxy-1,4-naphthoquinone, **III**) is a monohydroxy naphthoquinone pigment of ‘henna’[10a,b] plant possessing various biological properties, including antitumor activity[11]. It is also an effective chelator of divalent and trivalent metal ions due to its juxtaposed phenolic hydroxyl and keto groups[12-14]. The C-3 substituted derivatives of lawsone are particularly important since substitution at this position has been known to modulate its redox potential[15a,b], liposolubility[16] and the propensity of metal complexation[17,18]. To the best of our knowledge, the ability of the metal complexes of C-3 substituted lawsone derivatives to act as antitumor agents has so far not been examined. In the present report we describe synthesis, structural characterization and *in vitro* anticancer activities of a series of C-3 substituted phenylazo derivatives of lawsone and their copper complexes against the human breast cancer cell line MCF-7, which reveals enhanced antitumor activities for the metal complexes.

**Materials and Methods:**

All chemicals used in the syntheses of ligands and their metal complexes were of AR grade while solvents were distilled prior to their use. Lawsone (Sigma Chemicals), Toludines (Methyl Anilines)(Sisco Research Laboratories), $CuCl_2.2H_2O$ (Qualigens ), sodium nitrite (S. D. Fine chemicals Ltd. ) and sodium acetate (Merck) were used as supplied, except for the toluidines, which were purified further according to the literature methods[19].

Synthesis of ligands

Typically 0.3g (2.8 mmol) of the toluidine derivative was diazotised[20] using 0.19g (2.8 mmol) of sodium nitrite and 2N HCl. The diazonium salt was then allowed to couple with 0.5g (2.8 mmol) of lawsone in ethanol in the presence of excess sodium acetate at a temperature below 10°C. The precipitated orange-red coloured azo derivative was filtered, washed with cold water and ethanol and dried in vacuum. The crystals suitable for the X-ray diffraction studies were grown by slow evaporation of the acetonitrile solution of HMPAL2.

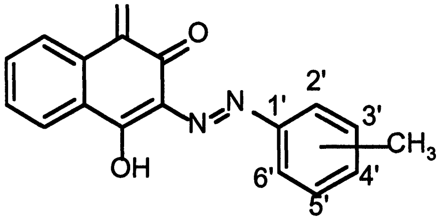

3-(2′- methyl phenylazo ) lawsone (HMPAL1); 3-(3′- methyl phenylazo ) lawsone (HMPAL2)
3-(4′- methyl phenylazo ) lawsone (HMPAL3)

**Figure 1:** Schematic representation of 3-(methyl phenylazo)lawsones (HMPAL)

Synthesis of Copper Complexes

The copper complexes were synthesized by refluxing the methanolic solutions of the ligands and copper chloride dihydrate in 2:1 molar ratio for 3 hours. The precipitated brown-red complexes were filtered, washed with cold ethanol and dried in vacuum.

Instruments

Elemental analyses were carried out using HOSLI CHN analyser at University of Pune. The molecular weights were determined by the Atmospheric Pressure Chemical Ionization Mass Spectroscopy (APCI-MS) carried out on a Hewlett-Packard 5989B quadrapole instrument connected to an electrospray 59987A unit with an APCI accessory and automatic injection using Hewlett-Packard 1100 series auto-sampler. Infrared spectra were recorded as KBr discs on a Mattson 3000 FTIR spectrophotometer. Proton NMR spectra were recorded in $CDCl_3$ on a Bruker AC 250 instrument operating at 250 MHz using $CDCl_3$ also as an internal reference standard. Electronic spectra were recorded on a Genesis-2 UV-VIS Spectrophotometer in the range 200-1100 nm. The magnetic susceptibility of the copper complexes were measured at 300K on a Faraday balance having field strength of 7000 KG using $Hg[Co(SCN)_4]$ as calibrant . Cycllic voltammetric measurements were made in DMF solvent on a Bioanalytical system BAS CV-27 with XY-recorder using platinum disc as working electrode against SCE and platinum wire as an auxiliary electrode with teraethyl ammmonium perchlorate (TEAP) as a supporting electrolyte.

X-ray crystal structure determination

All the measurements were made on a Siemens R3m/v diffractometer with Mo-Kα radiation (0.7107 Å). The data were collected at 293K on an orange crystal of 3-(3′-methyl phenylazo) lawsone (HMPAL2), $C_{17} H_{12} N_2 O_3$, having dimensions 0.35x 0.25x 0.25 mm. Full matrix least squares refinement of the setting angles (1.97°-25.01°) yielded a monoclinic cell with a = 6.930(2) Å, b = 4.851(2) Å, c = 20.782(8) Å,α= 90.00°,β= 95.38°, γ= 90.00°, V=695.5(4) $Å^{-3}$. For Z=2 and FW= 292.29, the calculated density was 1.396 $mg/m^3$. Based on the systematic absences, packing considerations, statistical analysis of the intensity distribution and the solution refinement of the structure, the space group was determined to be P21 /n. The structure refinement was done using SHELXL-93[21]. Of the 1334 reflections collected 1225 were unique ($R_{int}$ = 0,1012). Full matrix least squares refinements on $F^2$, gave weighted and unweighted agreement factors of $R_1$=0.0487, $WR_2$=0.1208 for $I > 2\sigma(I)$ and $R_1$=0.2160, $wR_2$=0.1740 for all data. Refinement on $F^2$ for all reflections except for three with very negative $F^2$ or flagged by the user for potential systematic errors, weighted R-factors wR and all goodness of fit S are based on $F^2$.Conventional R-factor R are based on F, with F set to zero for negative $F^2$. The observed criterion of $F^2 > 2\sigma(F^2)$ is used only for calculating R factor observed etc. and is not relevant to the choice of reflections for refinement. R-factors based on $F^2$ are statistically about twice as large as those based on F, and R-factors based on all data will be even larger. The molecule is distorted and occupies two semi-populated sites related by crystallographic inversion centres. Many atoms coincide and it was necessary to apply bond length constraints. Refinement using TWIN routine was unsuccessful confirming the distorted nature of the structure opposed to racemic twinning.

*Anticancer activity assay*

The breast tumor cell line, MCF-7, was maintained in RPMI culture medium (Gibco), containing penicillin (50U/ml) streptomycin (50 mg/ml) amphoteracin (2 mg/ml) and 10% fetal calf serum (Gibco). For experiments, cells seeded into 48 well culture plates at densities between 2000 to 5000 cells per $cm^2$, were kept at 37°C in an atmosphere of 5% $CO_2$. Test substances dissolved in DMSO (final concentration 0.1%) were added to cells 24h after seeding, and then cultures were maintained for a further 4 days. To determine live cell numbers, the dye 3-[4,5-dimethylthiazol-2yl]-2,5-diphenyltetrazolium bromide (MTT, Sigma Chemicals), a compound that is metabolized in the presence of the pyridine cofactors, NADH and NADPH, to give blue insoluble crystals, was added to cells for 4h (final concentration, 0.1 mg/ml). Crystals formed were then dissolved by the addition of a solution of sodium dodecyl sulphate (SDS) in HCl (final concentration of 10% and 10 milimolar respectively). Following the overnight solubilization of the blue crystals, colour intensity was read at 550 nm and values were recorded as absorbance units. Previously using this method, we have shown a good linear correlation between dye intensity and cell number[22].

**Results and discussion:**

The analytical and spectroscopic data on the ligands and their copper complexes are summarised in **Table 1.** Which clearly indicates that all copper complexes possess 1:2 metal to ligand stoichiometry. A perspective view of the HMPAL2 structure is shown in **Figure 2.**

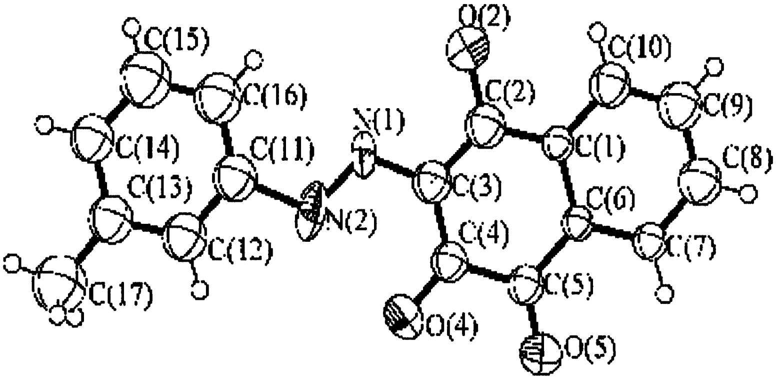

**Figure 2.** ORTEP plot of HMPAL2

Lawsone, with its ortho-hydroxy group is known to exhibit keto / enol tautomerism. Substitution at C-1 position have yielded essentially keto isomers as in case of 2-hydroxy-3-methyl-1-thiosemicarbazone-1,4 naphthoquinone[23]. In the present compound the bond lengths of C(2)-O(2) (1.36Å) and C(4)-O(4) (1.26 Å) confirm the existence of the keto form. The C(5)-O(5) bond distance is observed to be same as that in case of 2-hydroxy-3-methyl-1,4-naphthoquinone[34]. The elongated C(2)-O(2) bond is favourable for the intramolecular hydrogen bonding with the N(1)nitrogen of the azo linkage. The bond length of N(1)-N(2) linkage is characteristic of the azo form at least in the solid state[24] although azo / hydrazone tautomerism has been noted earlier for this series of compounds by Padhye et al.[25]. **Table 2** shows the selected bond lengths for HMPAL2 while **Table 3** gives the comparative data on the bond distances in related 1,4-naphthoquinones with C-3 substituted lawsone. It clearly indicates that the present compound crystallizes in a 1,2-naphthoquinone compound rather than as a 1,4 naphthoquinone moiety from a polar solvent like acetonitrile.

**Table 1:** Analytical data on the ligands and their copper complexes including electrochemistry, Mass-spectra, elemental analysis, magnetic moment and electronic spectra.

| Compound | Mass Spectral data APCI-MS $(M+H)^+$ | Elemental Analyses[a] %C %H %M | Electronic Spectra(nm)[b] | μ (300K) BM | $E_{1/2}$ in Volts. |
|---|---|---|---|---|---|
| HMPAL1 | 293 | 68.46 4.02 - (69.86) (4.11) | 210, 255, 429, 438 | - | -0.75[n],-1.45[r], -1.55[r] |
| HMPAL2 | 293 | 68.81 4.04 - (69.86) (4.11) | 207,258,426, 450 | - | -0.63[n] ,-1.42[r], -1.73[r] |
| HMPAL3 | 293 | 68.64 4.02 - (69.86) (4.11) | 210,258,426, 447 | - | -0.70[n]-1.26[r], -1.47[r] |
| $[Cu(MPAL1)_2]$ | 646 | 62.93 3.33 9.62 (63.20) (3.40) (9.84) | 406,523,643 [c] | 1.54 | -1.17[r],+0.32[r] |
| $[Cu(MPAL2)_2]$ | 646 | 63.10 3.49 9.69 (63.20) (3.40) (9.84) | 467,530,623 [c] | 1.58 | -1.28[r],+0.38[r] |
| $[Cu(MPAL3)_2]$ | 646 | 62.10 3.34 9.84 (63.20) (3.40) (9.84) | 461,563,632 [c] | 1.56 | -1.12[r],+0.37[r] |

[a]figures in parenthesis are calculated values, [b]In $CH_3CN$, [c]In Solid State as Nujol Mull, [n] non-reversible, [r] reversible.

**Table 2:** Inter-atomic distances (Å) in HMPAL2

| Distances (Å) | |
|---|---|
| C(2) - O(2) | 1.36 |
| C(5) - O(5) | 1.22 |
| C(4) - O(4) | 1.26 |
| C(3) - N(1) | 1.36 |
| N(1) - N(2) | 1.37 |
| N(2) - C(11) | 1.54 |
| C(13)-C(17) | 1.48 |
| C(1) - C(10) | 1.38 |
| C(6) - C(7) | 1.40 |
| C(11)-C(16) | 1.37 |

**Table 3:** Comparative data on bond distances of various 1,4-naphthoquinones

| Compound | Bond Distances (Å)[a] | | | Reference |
|---|---|---|---|---|
| | C(1) - O(1) | C(2) -O(2) | C(4) -O(4) | |
| 2-Hydroxy-1,4-naphthoquinone | 1.20 | 1.33 | 1.22 | 35 |
| 2-hydroxy-3-methyl-1,4-naphthoquinone | 1.22 | 1.36 | 1.22 | 36 |
| 2-hydroxy-3-chloro-1,4-naphthoquinone | 1.27 | 1.31 | 1.27 | 37 |
| 2-hydroxy-3-(3-methyl-2-butenyl)-1,4-naphthoquinone | 1.27 | 1.35 | 1.23 | 38 |
| 2-hydroxy-3-(3'-methylphenylazo)-1,4-naphthoquinone | 1.22 | 1.26 | 1.36 | Present work. |

[a] The numbering system adapted here is in accordance with the literature reports.

$^1H$ NMR data for the ligands indicates the absence of a signal due to the olefinic proton (δ 6.36 ppm) at C-3 position in lawsone[26] confirming the substitution of the azo chromophore at that position. A sharp singlet at δ2.4 ppm is due to the methyl protons on the arylazo ring while the multiplate observed at δ7.8 ppm is typical of the benzenoid protons of the quinone moiety[27].

**Table- 4** summarises the significant IR bands for the ligands and their metal complexes. The IR spectra of the 3-phenyl azolawsone derivatives show a broad hydroxyl absorption centred around 3400$cm^{-1}$ due to intramolecular hydrogen bonding between C-4 hydroxyl group and α- nitrogen of the azo linkage. This band is found to be absent in the copper compounds indicating the replacement of the hydroxyl proton by the metal. The two absorption bands occurring at 1695-1700cm$^{-1}$ and 1680-1684cm$^{-1}$ are typical of the 1,2-quinone carbonyl absorptions[26]. These bands do not exhibit any appreciable shift upon complexation suggesting that the carbonyls are not involved in metal comlexation. A medium absorption seen in all of the ligands around 1695cm$^{-1}$ is due to ν(-C=N-) stretch arising out of the hydrazinic C(3)-N(1) linkage due to tautomerization. The absence of this absorption in copper complexes confirms such an assignment. A weak absorption found around 1420-1450 cm$^{-1}$ is assigned to the unsymmetrical stretches for the IR inactive ν(-N=N-) linkage which is also observed in case of trans-p-substituted azobenzenes[28a] and arylazo naphthols[28b]. These bands undergo a shift to higher wavenumbers by 12-16cm$^{-1}$ upon metal complexation suggesting the involvement of azo nitrogen in chelation. However, it must be pointed out that these bands are quite weak being IR inactive. The band appearing around 968 cm$^{-1}$ associated with ν(C-N=N-C) stretching vibrations[29] is found to appear at higher wavenumbers upon metal complexation. The unaffected frequencies at 1590-1620cm$^{-1}$are assigned as the aromatic ν(C=C) vibrations.

**Table 4**. Significant IR bands for ligands and their copper complexes *

| Compound | ν(-OH) | ν(C=O) | ν(C=N) | ν(-C-N=N-C-) |
|---|---|---|---|---|
| HMPAL1 | 3420 | 1680, 1650 | 1700 | 966 |
| HMPAL2 | 3400 | 1683, 1650 | 1695 | 989 |
| HMPAL3 | 3425 | 1684, 1650 | 1698 | 969 |
| [Cu(MPAL1)$_2$] | - | 1685, 1663 | - | 976 |
| [Cu(MPAL1)$_2$] | - | 1682, 1656 | - | 997 |
| [Cu(MPAL1)$_2$] | - | 1681, 1666 | - | 978 |

* All values in cm$^{-1}$

The ortho-hydroxynaphthoquinones are known to exhibit an intense quinonoid absorption around 240-260 nm and a broad weak local excitation band around 420-430 nm respectively[26]. The C-3 substituted phenylazo lawsone derivatives described presently show similar absorptions with increased intensities for the band at 430-450 nm due to the extended conjugation. The electronic spectra for the copper complexes show a weak intensity band around 500-600 nm characteristic of dxy →dz$^2$ transition in a square planar copper environment [30a,,b]. Similar absorption has been noted in case of the square planar copper compound of 4,7-dimethyl-1,10-phenanthroline[31]. The magnetic moments of the present copper compounds at 300K fall in the range of 1.54-1.58 BM typical of square planar compounds undergoing antiferromagnetic exchange interactions due to stacking arrangements. Similar intermolecular spin coupling interactions have been observed by Pierpont et al.[32] in case of many ortho-quinone metal complexes. On the basis of the above structural data we would like to propose following tentative structure for the present copper compounds (**Figure 3**).

The cyclic voltammetric profiles of the ligand HMPAL2 and its copper complex in DMF are shown in **Figure 4** as a representative example of the present class of compounds. The redox potentials for other compounds are included in **Table 1**. It is observed that HMPAL2 ligand exhibits three reduction peaks at - 0.637, - 1.425 and -1.737 Volts respectively corresponding to the deprotonation, quinone to semiquinone and semiquinone to catechol conversion processes according to Bodini et al.[33] The latter two peaks are reversible while the former is irreversible. On complexation the deprotonation peak is found to be absent confirming the absence of hydroxyl group while the quinone to semiquinone peak is shifted to more positive potentials viz. -1.275 Volts maintaining its reversibility. The $Cu^{+2/+1}$ redox couple is observed in case of the copper complexes, centred at +0.32 to +0.38V. The peak corresponding to the semiquinone to catechol conversion is found to be absent in the copper complexes. The facile one-electron redox cycles observed for the copper compounds are known to generate reactive oxygen species (ROS) leading to cell damages[34a,b].

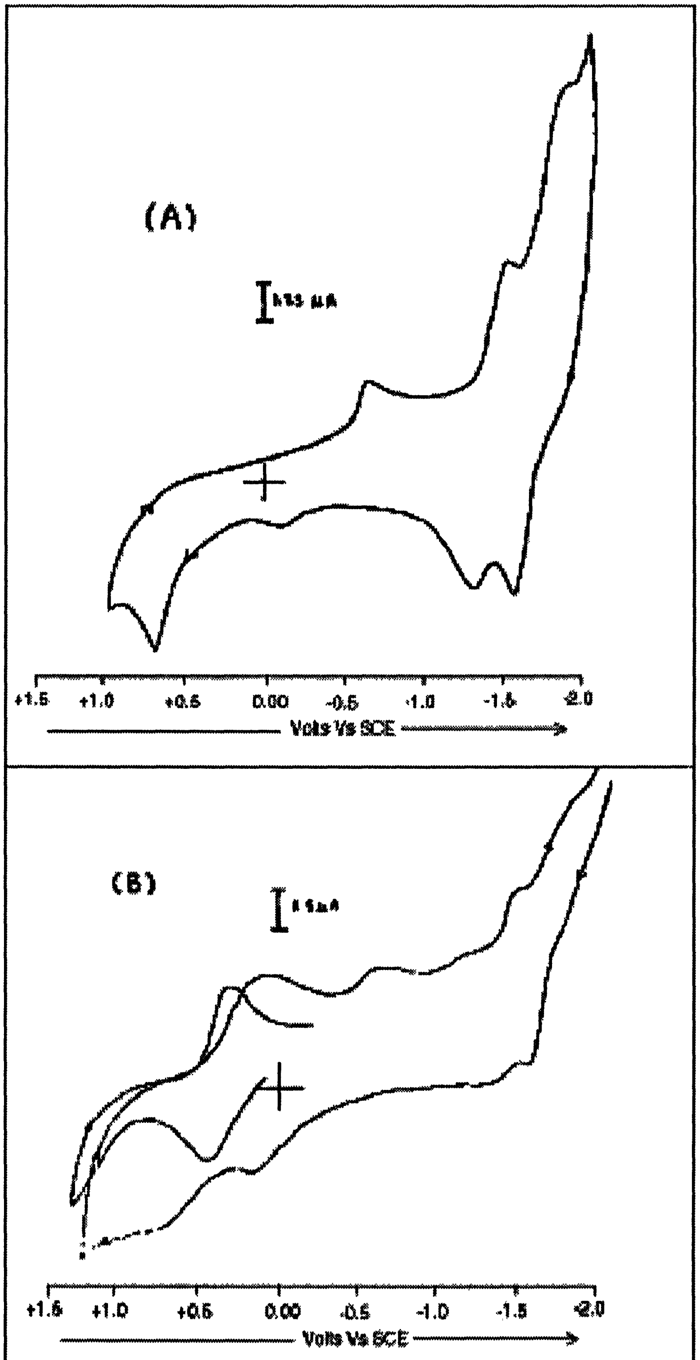

**Figure 4.** Cyclic voltammograms (100 mV/s) of a $10^{-3}$M solutions of (A) HMPAL2 (B) [Cu $(MPAL2)_2$] in (with reversible copper peak resolved inside) DMF.

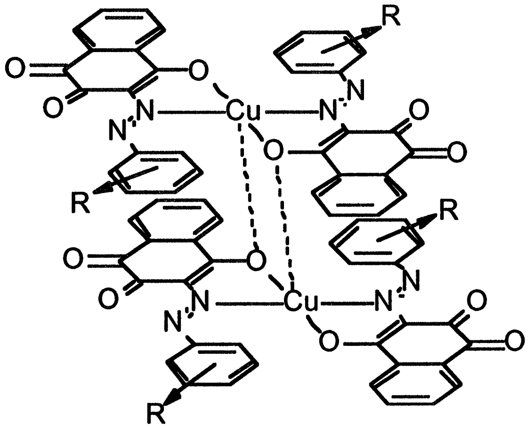

R=CH$_3$

**Figure 3**. Schematic representation of stacking of ligands in the copper complexes

All the compounds synthesized were tested on the human breast cancer cell line MCF-7. The antiproliferative activities of the copper compounds were observed at appreciably lower concentrations than their parent ligands (**Figure 5**) indicating that metal complexation with copper clearly offers an advantage in designing more effective compounds against the mammary tumours. Amongst the compounds tested [Cu(MPAL2)$_2$] shows the highest activity indicating that the *meta*-substitution on the arylazo ring is important for modulating electronic as well as steric parameters.

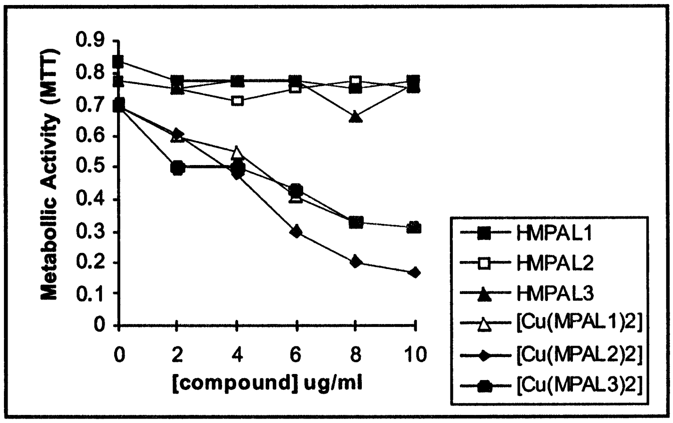

**Figure 5.** Cytotoxic effect of a series of phenylazo lawsone derivatives and their copper complexes.

**Acknowledgement**
SP and NG would like to thank the British Council for the HE Link Program. NG would like to thank Prof. David Billington, Dr. Dan Rathbone (Aston University, Birmingham), for kindly providing the instrumental facilities of MS, FTIR and NMR.

**References**

1. M. Miller, A. Rodgers, G. Cohen, *Biochem. Pharmacol.*, **35**, 1177, (1986).
2. H. Moore, *Science*, **197**, 527, (1977).
3. H. Kappus, *Biochem. Pharmacol.*, **35**,1,(1986)
4. T. Gannt, D. Rao, R. Mason, R. Cohen, *Chem. Biol. Interact.*, **65**, 157, (1988)
5. Y. You, X. Zheng, R. Young, B. Ahn, *Archiv. Pharmacol. Research*, **21**, 595, (1998)
6. P. D'Arpa, L. Liu, *Biochem. Biophys. Acta*, **989**, 163, (1989)
7. N. Fujji, Y. Arima, Y. Yamashita, M. Nagashima, H. Nakona, *Antimicrob. Agents Chemother.*, **36**,2589,(1992).
8. O. Moullet, J. Dreyer, *Biochem. J.*, **300**, 99, (1994).
9. Z. Plyta, T. Li, V. Papageorgiou, A. Mellidis, A. Assimopoulou, E. Pitsinos, E. Culadouros, *Bioorg.*

*Med. Chem. Lett.*, **8**, 3385, (1998).
10. (a) A. Leung, S. Foster, *Encyclopedia of Common Natural Ingredients used in food, drugs and cosmetics,* **2nd** Edn., John Wiley & Sons., Inc.
(b) B. Ali, A. Bashir, M. Tanira, *Pharmacology,* **51**,356, (1995).
11. J. Duke, *Medicinal Plants of the Bible*, Trado-Medic Books, (1983).
12. A. Kumbhar, S. Padhye, D. Ross, *Biometals*, **9**, 235, (1996).
13. V. Kelkar, R. Gokhale, H. Gholap, *Synthesis React. Inorg. Metal Org. Chem.*, **28**, 1253, (1998).
14. V. Kelakar, R. Gokhale, *Polish J. Chem.*, **72**, 655, (1998).
15. (a) Fieser and Fieser, *J. Am.Chem.Soc.*,**56**,1565,(1934).
(b) Ball , *J. Biol. Chem.*, **106**,515,(1934),**114**,649,(1936)
16. L.Fieser, M. Ettinger, G. Fawaz, *J. Am.Chem.Soc.*, **70**, 3228
17. S.Padhye, P. Garge, M. Gupta, *Inorg. Chim. Acta.*,**152**, 37, (1988)
18. R. Chikate, A. Bajaj, A. Kumbhar, V. Kolhe, S. Padhye, *Thermochimica Acta*, **249**, 239, (1995).
19. D. Perrin, E. Armarego, D. Perrin, *Purification of Laboratory Chemicals*, Pergammon Press, (1966)
20. B. Furniss, J. Antony, P. Hannaford, G. Smith, A. Tatchell, *Vogel's Textbook of Practical Organic Chemistry*,**5th** Edn. , ELBS, Longman, (1989).
21. G. Sheldrick, SHELXL-93, Program for Crystal Structure Determination, University of Gottingen, 1993.
22. C.Newton, *J. Steroid Biochem. Molec. Biol.,*. **55**, 327, (1995)
23. D. Saha, J. Patole, S. Padhye, E. Sinn, Unpublished data.
24. W. Cross, K. Flower, R. Pritchard, *J. Chem.Research,* **(s)**, 178, (1999) and references therein.
25. S. Padhye, *J. Univ. Poona, Sci. Tech.* 52: 49-56, (1979)
26. R. Thompson, *Naturally Occurring Quinones,* **2nd** Edn. , Academic Press, {1971).
27. Aldrich Library for 1H&13C NMR ,2nd Edn., 2, **86A**.
28. (a) R. Silverstein, G. Bassaler, T. Morill, *Spectroscopic Identification of Organic Compounds*, **5th** Edn. , John Wiley & Sons Inc.
(b) K. Morgan, *J. Chem. Soc.*, 2151, (1961)
29. K. Tetlow, *Research*, **3**, 187, (1950).
30. (a) S.M. Nelson, *Pure appl. Chem.* ,**52**, 2461,(1980)
(b) B.J. Hathaway, M. Duggan, A . Murphy, J. Mullane, C. Power, A. Walsh and B. Walsh, *Coord. Chem. Rev.*, **36**, 267, (1981).
31. C. Su, T. Tai, S.Wu, S. Wang, F. Liao, *Polyhedron*, **18**, 2361,(1999)
32. (a) C. G. Pierpont and R. M. Buchanan, *Coord. Chem. Rev.*, **38**, 45, (1981) and references therein.
(b) C. G. Pierpont and C.W. Lange,*Coord. Chem. Rev.*, **41**, 334, (1994) and references therein.
33. M. E. Bodini, P. E. Bravo, V. Arancibia. M., *polyhedron*, **13**, 497, (1994)
34. (a) C. Newton , N. Drummond , C. Burgoyne, V. Speirs , G. Stalla, S. Atkin *J Endocrinol.*, **161**, 199, (1999).
(b) P.A. Thibodeau, B. Paquette, *Free Radical Biology and Medicine*, **27**, 1367,(1999).
35. J. Dekkers, H. Kooijman, J Kroon, E.Grech, *Acta Cryst.*, **C50**, 2896, (1996).
36. J. Gaultier, C. Hauw, *Acta Cryst.* , **19**, 919, (1965).
37. J. Gaultier, C. Hauw, *Acta Cryst.* , **19**, 580, (1965).
38. .Larson, L. Anderson, B. Pedersen, *Acta Cryst.*, **C48**, 2009, (1992)

**Received: January 18, 2000 - Accepted: January 26, 2000 - Received in publishable format: May 5, 2000**

